# Hepatectomy in a case of hepatocellular carcinoma with constitutional indocyanine green excretory defect

**DOI:** 10.1016/j.ijscr.2018.10.074

**Published:** 2018-11-01

**Authors:** Richi Nakatake, Morihiko Ishizaki, Chika Miyasaka, Kosuke Matsui, Masaki Kaibori

**Affiliations:** a Department of Surgery, Kansai Medical University, 2-5-1, Hirakata, Osaka 573-1010, Japan; b Department of Pathology and Laboratory Medicine, Kansai Medical University, 2-5-1, Hirakata, Osaka 573-1010, Japan

**Keywords:** ICG, indocyanine green, ICGR15, indocyanine green retention rate at 15 min, GSA, ^99m^Tc-galactosyl human serum albumin, CT, computed tomography, S, segment, (PET)-CT, positron emission tomography, HCC, hepatocellular carcinoma, CP, Child–Pugh, LHL15, liver scintigraphy, HH15, heart uptake ratio, GSA-Rmax, maximal removal rate of ^99m^Tc-GSA, GSA-RL, GSA-Rmax in the predicted residual liver, Constitutional indocyanine green excretory defect, ^99m^Tc-galactosyl human serum albumin (GSA), Liver resection, Hepatocellular carcinoma

## Abstract

Introduction: Constitutional indocyanine green (ICG) excretory defect is extremely rare. The indocyanine green retention rate at15 min (ICGR15) is important for estimating hepatic functional reserve and selection of the appropriate surgical procedure before hepatectomy is performed. Because of the rarity of constitutional ICG excretory defect, its clinical features are not well understood. We report here evaluation and treatment of a patient with such a disorder.

Presentation of case: An 83-year-old man was admitted to hospital with the diagnosis of resectable hepatocellular carcinoma. The preoperative indocyanine green (ICG) retention rate at 15 min was greater than 76.2%. Despite this finding, Child–Pugh classification and ^99m^Tc-galactosyl human serum albumin (GSA) liver scintigraphy didn’t show any abnormal findings, and there was no background disease. Therefore, we diagnosed him with constitutional ICG excretory defect and performed partial hepatectomy. For patients requiring hepatectomy with this disease the indications and procedure for surgery should be considered. These should be based on liver function tests such as GSA liver scintigraphy.

Conclusions: Constitutional ICG excretory defect is an extremely rare disorder. At present, the indications for surgery for this condition should be comprehensively considered. Findings of liver function tests, such as a general liver function test and GSA liver scintigraphy, are important for treating this disorder.

## Highlights

• Constitutional indocyanine green excretory defect is extremely rare.
• The ICGR15 is important for estimating hepatic functional reserve and selection of the appropriate surgical procedure.
• Because of the rarity of constitutional ICG excretory defect, its clinical features are not well understood.
• At present, the indications for surgery for this condition should be comprehensively considered.
• Findings of liver function tests, such as a general liver function test and GSA liver scintigraphy, are important for treating this disorder.

## 1 Introduction

Constitutional indocyanine green (ICG) excretory defect is extremely rare. Only five reports of hepatectomy in patients with a constitutional ICG excretory defect have been published in the English language literature until 2017 (Table 1) [[1], [2], [3], [4], [5]]. Loss of active ICG transport across the hepatic membrane is thought to be the cause of this disorder [6,7]. Because of advances in preoperative assessment of liver function, liver resection is a relatively safe procedure. The indocyanine green retention rate at 15 min (ICGR15) is important for estimating hepatic functional reserve and selection of the appropriate surgical procedure before hepatectomy is performed. Because of the rarity of constitutional ICG excretory defect, its clinical features are not well understood. We report here evaluation and treatment of a patient with such a disorder. This work has been reported in line with the SCARE criteria [8].

**Table 1:** Previously reported cases of hepatectomy with constitutional indocyanine green excretory defect.

Author	Year	Age/sex	ICG R15	Child-Pugh grade	Disease	Preoperative liver functional evaluation	HH15/LHL15	Operation	Postoperative complications
Hanazaki et al.	2000	47/F	59.8	N.D	Cavernous hemangioma	GSA liver scintigraphy	0.49/0.86	Left lateral sectionectomy	none
Yamanaka et al.	2001	61/M	72	A	HCC	GSA liver scintigraphy,liver biopsy	0.54/0.94	Partial hepatectomy (S8)	none
Kadono et al.	2006	78/F	79.3	A	Bile duct cystadenocarcinoma	GSA liver scintigraphy, AKBR	N.D/0.96	Left hepatectomy	none
Maeda et al.	2007	69/F	83.8	A	HCC	BTR	none	Right anterior sectionectomy	none
Aoki et al.	2013	77/M	77.1	B	HCC	GSA liver scintigraphy	0.53/0.89	Left medial sectionectomy + resection of the ventral region of the anterior segment	hyperbilirubinemia
Our case		83/M	76.2	A	HCC	GSA liver scintigraphy	0.482/0.931	Partial hepatectomy (S4)	none

HCC:hepatocellular carcinoma, AKBR: arterial ketone body ratio, GSA: 99 mTc-galactosyl-human serum albumin, BTR: branched chain amino acid and tyrosine ratio, N.D: not described.

## 2 Case presentation

**Fig. 1:**
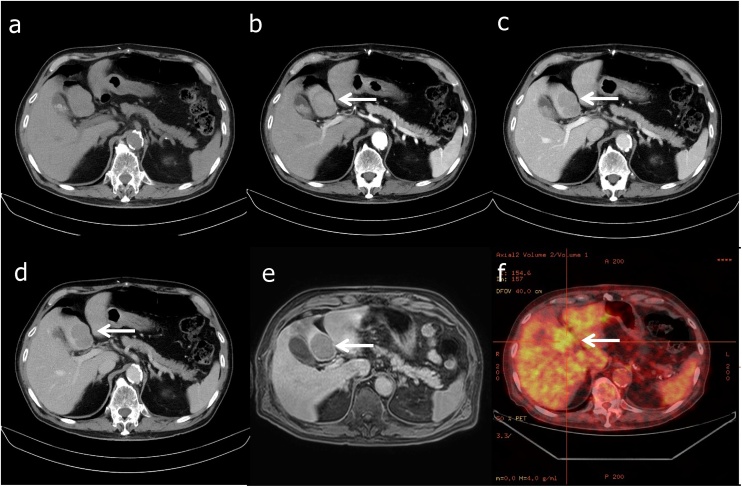
(a–d) Liver dynamic computed tomography (CT). (a) Plain CT, (b) arterial phase, (c) portal phase and (d) delayed phase (arrows). The tumor, located in the segment 4 of the liver, measured 40 mm in diameter. The density of the tumor was well enhanced in the arterial phase and washed-out in the portal phase. (e) The hepatobiliary phase of Gd-EOB-DTPA-MRI shows tumor nodules in the liver with low intensity in segment 4 (arrow). (f) Positron emission tomography-CT. The SUV max of the tumor in S4 of the liver is 3.2 (arrow).

An 83-year-old man was admitted to our hospital for evaluation and management of a symptomatic liver mass. His medical history included diffuse large B-cell lymphoma, which was treated with rituximab + pirarubicin + cyclophosphamide + vincristine + prednisone therapy at 81 years old, and had bladder cancer (resected at 67 years) on follow-up. After resection of the bladder cancer, no recurrence was detected for 16 years. Liver dynamic computed tomography (CT) showed a low-density mass in the segment (S) 4 area, measured 40 mm in diameter. The density of the tumor was well enhanced in the arterial phase and washed-out in the portal phase. (Fig. 1a–d). The hepatobiliary phase of Gd-EOB-DTPA-MRI shows tumor nodules in the liver with low intensity (Fig. 1e). On positron emission tomography (PET)-CT, the maximum standard uptake value of the tumor in S4 of the liver was 3.2 (Fig. 1f). MRI and PET-CT confirmed a single liver tumor that was 40 mm in diameter and located in the S4 region. Liver metastasis of malignant lymphoma was suspected because of the patient’s medical history. Therefore, we performed a liver biopsy preoperatively. The patient was diagnosed with hepatocellular carcinoma (HCC) based on the biopsy results and imaging findings.

Upon presentation, the patient was afebrile, had no history of weight loss, and his appetite was good. His height was 166 cm, body weight 72 kg, and BMI 26.12. He has no drinking history. In a preoperative indocyanine green (ICG) test, the ICGR15 was 76.2%. The total bilirubin level was 1.1 mg/dL and the direct bilirubin level was 0.2 mg/dL. The serum albumin level was 4.7 g/dL and prothrombin activity was 96.3%. The Child–Pugh (CP) score was 5 points, which indicated a grade of A. The degree of liver damage was equivalent to A in accordance with the scoring system of the Liver Cancer Study Group of Japan. Table 2 shows the patient’s laboratory data on admission. The hepatic uptake ratio of ^99m^Tc-galactosyl human serum albumin (GSA) by liver scintigraphy (LHL15) was 0.931 and the heart uptake ratio (HH15) was 0.482. The maximal removal rate of ^99m^Tc-GSA (GSA-Rmax) was 0.874 mg/min. GSA-Rmax in the predicted residual liver (GSA-RL) was greater than 0.765 mg/min, which was within the range considered safe for surgical procedures.

**Table 2:** Laboratory data on the initial visit.

WBC	4100/μl	AFP	2 ng/ml
RBC	421/μl	CA19-9	28.3 U/ml
Hb	12.9 g/dl	CEA	5.2 ng/ml
Plt	15.6 μl	PIVKA-II	92 U/ml
PT	91.4%		
APTT	29 sec	HBs-Ag	(−)
TP	6.8 g/dl	HBs-Ab	(−)
Alb	4.7 g/dl	HBc-Ab	(−)
BUN	12 smg/dl	HCV-Ab	(−)
Cre	0.84 mg/dl		
Na	143 mmol/l	ANA	(−)
K	3.8 mmol/l	AMA	(−)
Cl	106 mmol/l		
AST	32 U/l	ICG R15	76.2
ALT	24 U/l		
ALP	311 U/l	99mTc-GSA	
LDH	339 U/l	LHL15	0.931
T-Bil	1.1 mg/dl	HH15	0.482
D-Bil	0.2 mg/dl	LHL/HH	1.932
γ-GTP	46 U/l		
ChE	282 U/l	GSA-Rmax (mg/min)	
CRP	0.032 mg/dl	Total	0.874
		Anterior segment	0.313
		Posterior segment	0.267
		Lateral segmental＋caudate lobe	0.185
		Medial segment	0.109

**Fig. 2:**
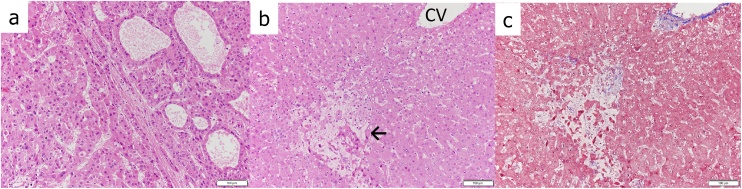
Microscopic findings (hematoxylin–eosin staining, ×200). (a) Cancerous area and (b) non-cancerous area. Expansion of perisinusoidal cells and an atrophic hepatic cord in the background of liver tissue can be seen (arrow). Fatty changes were observed in some areas. (c) Manson trichrome staining (×200) shows that there is no fibrosis.

Despite this finding, Child–Pugh classification and ^99m^Tc-GSA liver scintigraphy did not show any abnormal findings, and there was no background disease. Antibody against hepatitis C virus and hepatitis B virus surface antigen were negative. The serum anti-mitochondrial antibody and anti-nuclear antibody were negative. The serum tumor markers alpha-fetoprotein, carcinoembryonic antigen, and cancer antigen 19-9 were within the and normal range, but the protein level induced by vitamin K absence-II levels was increased (92 mg/dL). Therefore, we diagnosed constitutional ICG excretory defect with HCC and decided to perform radical surgery. Therefore, the patient underwent partial hepatectomy (S4). Pathologically, the tumor was diagnosed as moderately differentiated HCC (Fig. 2a). There was expansion and bleeding of perisinusoidal cells and an atrophic hepatic cord in the background of liver tissue. Because of previous chemotherapy, the diagnosis of sinusoidal obstruction syndrome (SOS) of the liver was established (Fig. 2b). After partial hepatectomy (S4), the postoperative course was uneventful and the patient was discharged on the 8th postoperative day. The patient remains in good general condition.

## 3 Discussion

Hepatectomy in cases of constitutional ICG excretory defect is exceedingly rare. Only five reports of hepatectomy with this defect have been reported. Among these cases, only three patients had HCC [2,4,5]. Two other cases showed cavernous hemangioma and biliary cystadenocarcinoma [1,3]. All of the patients were Japanese. The postoperative course in these patients was uneventful, except for only one patient with liver cirrhosis who also suffered from hyperbilirubinemia [5].

To the best of our knowledge, constitutional ICG excretory defect has only been reported in Japan. The ICG test is not usually performed in countries other than Japan and this disorder does not show any clinical symptoms. Therefore, unless the ICG test is frequently carried out on a regular basis, this disorder will likely not be observed. In Japan, the ICG test is considered one of the most important preoperative factors for estimation of hepatic functional reserve [[9], [10], [11]]. For assessment of patient hepatic functional reserve, the ICGR15 is an important factor, but it was outside the normal range in our case. Because variability in ICG values depends on hepatic blood flow, parenchymal cellular function, and biliary excretion, ICGR15 values are not reliable in the case of jaundice, the presence of a port-systemic shunt, or an ICG excretory defect [12]. Therefore, there is a problem of determining what the next step should be for evaluating patients.

GSA liver scintigraphy has been hypothesized to be the best modality with which to evaluate hepatic functional reserve [4]. Because this agent binds to hepatocytes for a long period, the distribution of the functioning hepatocyte mass can be assessed by performing single-photon emission computed tomography with ^99m^Tc-GSA [13]. Significant correlations have been observed between ICGR15 and both LHL15 (a receptor index) and HH15 (an index of blood clearance) (12). GSA-Rmax, which is calculated by using a radiopharmacokinetic model, is also correlated with the severity of liver disease. There is a significant difference in GSA-Rmax between patients with chronic hepatitis and normal liver function [14]. GSA-Rmax is useful for selecting candidates for hepatectomy. Extended hepatectomies are high-risk surgical procedures in the case of low GSA-Rmax scores (<0.35) [14]. GSA-RL should be maintained at greater than 0.15 to avoid postoperative hyperbilirubinemia or hepatic failure [15]. Aoki et al. reported that patients with Dubin–Johnson syndrome and an ICG excretory defect should be analyzed by GSA scintigraphy for safe and successful hepatectomy procedures [5]. GSA scintigraphy showed a more accurate hepatic functional reserve in our case, which is why we used it to evaluate the predictive score.

In our case, liver injury included histological changes as SOS may be correlated to administration of cyclophosphamide as side effect [16]. A correlation between ICGR15 values and SOS has been described [17,18]. The cut-off point of the ICGR15 test that correlated with diagnosis of SOS was 8% [19]. Although the background of liver tissue was mild SOS pathologically, the ICG R15 value was extremely high. Furthermore, GSA-Rmax was within the normal range. We finally concluded that the high value of the ICGR15 was affected by constitutional ICG excretory defect rather than SOS.

## 4 Conclusion

In conclusion, constitutional ICG excretory defect is an extremely rare disorder. At present, the indications for surgery for this condition should be comprehensively considered. Findings of liver function tests, such as a general liver function test and GSA liver scintigraphy, are important for treating this disorder.

## Conflicts of interest

All authors have no conflict of interest to disclose.

## Funding

All authors have no funding to disclose.

## Ethical approval

Our institution exempts ethical approval for case report.

## Consent

Written informed consent was obtained from the patient for publication of this case report and accompanying images. A copy of the written consent is available for review by the Editor-in-Chief of this journal on request.

## Author’s contributions

RN drafted the manuscript. MK has given the final approval of the version to be published. All authors read and approved the final manuscript.

## Registration of research studies

This is not a research article.

## Guarantor

Richi Nakatake.

e-mail: nakatakr@hirakata.kmu.ac.jp.

Department of Surgery, Kansai Medical University, 2-5-1 Shinmachi, Hirakata, Osaka 573-1010, Japan.

## Provenance and peer review

Not commissioned, externally peer reviewed.
